# The Cutaneous Microbiome and Wounds: New Molecular Targets to Promote Wound Healing

**DOI:** 10.3390/ijms19092699

**Published:** 2018-09-11

**Authors:** Taylor R. Johnson, Belinda I. Gómez, Matthew K. McIntyre, Michael A. Dubick, Robert J. Christy, Susannah E. Nicholson, David M. Burmeister

**Affiliations:** 1 Department of Surgery, The University of Texas Health Science Center at San Antonio, 7703 Floyd Curl Dr., San Antonio, TX 78229, USA; JohnsonTR@livemail.uthscsa.edu (T.R.J.); michael.a.dubick.civ@mail.mil (M.A.D.); nicholsons@uthscsa.edu (S.E.N.); 2 United States Army Institute of Surgical Research, 3650 Chambers Pass, JBSA Fort Sam Houston, TX 78234, USA; belinda.i.gomez.ctr@mail.mil (B.I.G.); mkm3@bu.edu (M.K.M.); robert.j.christy12.civ@mail.mil (R.J.C.); 3 School of Medicine, New York Medical College, Valhalla, New York, NY 10595, USA

**Keywords:** skin, wound healing, microbiome, infection, antibiotic resistance, commensals

## Abstract

The ecological community of microorganisms in/on humans, termed the microbiome, is vital for sustaining homeostasis. While culture-independent techniques have revealed the role of the gut microbiome in human health and disease, the role of the cutaneous microbiome in wound healing is less defined. Skin commensals are essential in the maintenance of the epithelial barrier function, regulation of the host immune system, and protection from invading pathogenic microorganisms. In this review, we summarize the literature derived from pre-clinical and clinical studies on how changes in the microbiome of various acute and chronic skin wounds impact wound healing tissue regeneration. Furthermore, we review the mechanistic insights garnered from model wound healing systems. Finally, in the face of growing concern about antibiotic-resistance, we will discuss alternative strategies for the treatment of infected wounds to improve wound healing and outcomes. Taken together, it has become apparent that commensals, symbionts, and pathogens on human skin have an intimate role in the inflammatory response that highlights several potential strategies to treat infected, non-healing wounds. Despite these promising results, there are some contradictory and controversial findings from existing studies and more research is needed to define the role of the human skin microbiome in acute and chronic wound healing.

## 1. Introduction

The human microbiome is a vital component in both the maintenance of human health and the establishment of human disease. The number of all microbial cells that inhabit the human body outnumbers host cells by a factor of 10, while the number of genes is eclipsed by a factor of 100. Next generation sequencing methods have allowed us to link alterations in the gastrointestinal microbiome to the pathogenesis of autoimmune, metabolic, and atopic diseases [1,2,3,4]. Despite the fact that similar proportions of host to microbial cells exist on the cutaneous microbiome [5], the impact of the cutaneous microbiome on acute and chronic wound healing is less defined.

Both in-vivo and in-vitro studies of the cutaneous microbiome have supported a general consensus that the microbial composition of skin wounds impacts wound healing. However, the conclusions drawn from these studies have been conflicting. Canesso et al. demonstrated that in the absence of commensal skin microbiota, Swiss mice demonstrated accelerated wound closure and epithelization with a significantly altered wound leukocyte profile [6]. In addition, Germ-Free mice demonstrated increased levels of anti-inflammatory cytokines, upregulation of vascular endothelial growth factor (VEGF), and decreased scar formation [6]. When the skin microbiota were restored, their wound healing profiles were similar to that of conventional mice, suggesting that commensal bacteria may impede wound healing. In contrast, oral Vancomycin-treated rodents were found to have decreased bacterial density of skin wounds and slower wound healing rates, potentially due to the downregulation of proteins interleukin (IL)-17 and regenerating islet derived protein-III gamma (RegIIIy) which are essential for keratinocyte differentiation and proliferation [7].

These conflicting studies suggest that the impact of the microbiome on wound healing is multifaceted, and a balance between types of organisms (e.g., viruses vs bacteria, Gram Negative vs. Gram Positive, etc.) is needed to promote skin health and regeneration. Simple reductions in bacterial burden achieved through anti-microbial treatments may reduce beneficial bacteria, and alternative strategies should be considered, especially considering the concerns over antibiotic resistance. In this review, we analyze the current literature regarding the impact of the skin microbiome on wound healing. We will start by discussing the constituents of the normal human microbiome and how it is altered in acute and chronic wounds. We will then consider preclinical studies that include both animal and in vitro model systems to identify molecular targets that may be leveraged to promote wound healing and tissue regeneration. Finally, we discuss the question: Can the microbiome be modulated or changed to promote optimal wound healing and improve patient outcomes?

## 2. Defining the Healthy Skin Microbiome

### 2.1. Bacteria

An individual’s skin microbiota is established intra-partum, with maternal delivery playing a vital role in microbial composition [8]. Site specific colonization, demonstrated by microbial communities residing in distinct epidermal topographical niches, is a key feature of the human skin microbiome [9,10]. It is now apparent that at least 19 bacterial phyla and over 1000 bacterial species have been identified within the most superficial level of skin [9,11]. The most commonly represented epidermal bacterial phyla include *Actinobacteria* (52%), *Firmicutes* (24%), *Proteobacteria* (17%), and *Bacteroidetes* (7%), whereas the most commonly represented genera (phyla) include *Corynebacteria* (*Actinobacteria*), *Propionibacteria* (*Actinobacteria*) and *Staphylococci* (*Firmicutes*) [9,11]. Microbial composition varies across skin sites, both due to moisture content and anatomical location. For example, sites containing many sebaceous glands (e.g., glabella, alar crease, external auditory canal, retroauricular crease, occiput, manubrium, and back) often contain the greatest bacterial load [12] and are mainly dominated by *Propionibacterium* and *Staphylococci* spp., whereas moist sites are dominated by *Corynebacteria* and *Staphylococci* spp. [9]. Dry sites (e.g., volar forearm, hypothenar palm and buttock), despite demonstrating the greatest microbial diversity and variability overall, contain a greater abundance of *β-proteobacteria*, *Flavobacteriales*, and other Gram Negative organisms [9,13]. In addition to epidermal colonization, recent literature has revealed that the healthy skin microbiome extends into the sub epidermal compartments with higher proportions of *Proteobacteria* (*Burkholderiales* and *Pseudomonadales* species) and *Actinobacteria* and a lower abundance of *Firmicutes* [14].

### 2.2. Fungi

Less commonly defined is the fungal component of the microbiome (i.e., mycobiome). Interestingly, some studies have shown that (in contrast to bacteria) fungal species often differ by anatomical location (head, torso, arm, leg, and feet) independently of moisture or sebaceous content [15]. While cultivation methods in earlier studies have shown that the *Malassezia* genus is the major component of the skin fungal community, sequencing of 18S rDNA in healthy patients has confirmed that *Malassezia* organisms (which includes some known pathogens) dominate the mycobiome on most skin sites [16,17], with colonization of the feet being an exception. The increased display of fungal diversity and lower stability of fungal organisms in this area might explain why diseases of the heel, toe web, and toenail are common sites of recurrent fungal infections.

### 2.3. Viruses

Methods to characterize the human skin microbiome have mainly focused on targeting bacterial and fungal signatures through ribosomal DNA-based amplification. Given the lack of such sequences in virus and bacteriophage genomes along with the low relative abundance of viruses in the skin, amplicon detection of the viral/phage microbiome via standard methods remains challenging [18,19,20]. In addition, the occurrence of “Viral Dark Matter” defined as metagenomic sequences originating from viral genomes that have not been aligned with their host microbes are a major obstacle in comprehensively defining the skin virome [21]. However, most recently, high throughput metagenomic sequencing and polymerase chain reaction (PCR) quantification have identified the Human Papilloma Virus (*Papillomaviridae* (HPV)) as one of the most common species within the healthy skin virome [22,23,24]. In addition, the Human Polyomavirus and Circoviruses are main components of the skin virome identified through the use of whole metagenomic analysis [25]. Moreover, bacteriophages are also major components of the skin virome. *Staphylococcus* phages were found to contain hypervariable loci in the virome of healthy human volunteers, whereas *Propionibacterium* phages were found to be minimally divergent. The abundance of these bacteria infecting microbes has allowed us to identify them as potential targets in wound healing; recent potential therapeutic targets for adult acne have included phage-based therapies [26]. Likewise, more studies are needed to elucidate the impact of vaccinations combating viral pathogens on skin viral commensals. For example, a recent case report found that widespread HPV-2 positive warts completely regressed following administration of HPV vaccination in a young boy [27].

### 2.4. Other Factors Defining the Skin Microbiome

While much of the research characterizing the healthy human skin microbiome focuses on anatomical locations and water/oil content, it has been shown that genetics and environmental factors such as climate also help define the normal flora [28]. For example, skin commensals of the forearms of Venezuelans (dominated by *Staphylococcus* and *Proteobacteria*) differed considerably from that of Americans (dominated by *Propionibacterium*) [29]. Moreover, in addition to age and gender, the microbiome can predict whether inhabitants live in an urban or rural area within the same metropolitan area [30]. Similarly, Hospodsky et al. demonstrated that the hands of Tanzanian women had a greater abundance of soil associated microbes such as *Rhodobacteraceae* and *Nocardioidaceae* compared to women in the United States [31]. Lastly, Leung et al. introduced the concept of the pan-microbiome to suggest that the microbial members of the skin microbiome varied across countries, with specific differences in Chinese individuals when compared to other racial groups (e.g., Americans, Tanzanians) [32]. Because differences in skin commensals may affect progress in wound healing and subsequent treatment, the above ethnic and environment related differences underscore the need to expand current knowledge to more diverse geographic and cultural populations.

## 3. The Cutaneous Microbiome: Aberrations in Human Wounds

Wound healing is a multi-layered process consisting of sequential yet overlapping phases that begin as an inflammatory response to the physical disruption of tissue [33]. Indeed, because wound healing is intimately tied in with inflammation and immune cells [34], much of the molecular information on the effects of commensal and pathogenic bacteria are tied to inflammatory signaling. However, comparatively speaking, there are many more studies that examine wound healing histologically than there are molecular analyses. While the wound healing process has been characterized extensively, the burgeoning field of microbiome analysis is just beginning to be understood. As summarized below, many studies have concentrated on the relative amounts of different bacterial flora, such as identifying the major phyla, genus, and so on, located within different types of wounds. However, determination of overall bacterial diversity that leads to skin dysbiosis may be more revealing [35]. Using the gut as an example, both alpha-diversity (microbial variety within a given population) and beta-diversity (microbial variety between populations) have been differentially affected by factors such as diet [36] and may have implications for chronic disease development [37]. For the skin, treatment of atopic dermatitis with emollients was shown to improve symptoms, increase microbial diversity, and decrease *Staphylococcus* abundance [38]. In terms of wounds, the study of diversity has largely been limited to diversity of biofilms that form in chronic wounds [39], which will be discussed first. However, it should be noted that because much of the focus has been on bacterial burden and specific organism changes, there remains a considerable amount to discover regarding the role of altered diversity measures in wound healing.

### 3.1. Chronic Wounds

Chronic, hard-to-heal wounds such as diabetic foot ulcers (DFU), decubitus ulcers (DU), venous leg ulcers (VLU), and post-surgical wounds generate a major burden not only for the patient and their provider, but also for the healthcare system; it is estimated that the treatment of these chronic wounds accounts for up to 25 billion US dollars annually [40]. Polymicrobial biofilms, which foster pathogenic microbial growth and disrupt the coordinated events of wound healing, are highly abundant in chronic wounds and play a vital role in the pathogenesis of impaired cutaneous healing [41,42]. A clinical study examining 50 chronic wound specimens from adult patients revealed a greater prevalence in biofilm production in 60% of chronic wounds compared to only 6% of acute wounds [41]. In addition, Wolcott et al. identified *Pseudomonas* as not only the most dominate genera in chronic wound biofilms, but also the most common microbe seen in biofilms formed from a single species [42]. Biofilms form as a result of microbial aggregation on a surface, encased in an exo-polymeric substance consisting of polysaccharides, lipids, and protein [43]. Through this matrix-type substance, microbes are able to alter their proliferative rate, metabolic activities [44], and develop a phenomenon known as quorum sensing, which is the ability for microbes to communicate changes in response to population density through the production of organic signaling molecules [45]. Biofilms provide continuous stimulation to the innate immune system, which ultimately delays progression to the proliferative phase of wound healing.

The microbial composition of human skin is not static, and the presence and abundance of microbes in skin wounds depend on wound type. The three main phyla identified in pressure ulcers are similar to that of healthy commensals (e.g., *Firmicutes*, *Proteobacteria*, and *Actinobacteria*) [46]. In a large clinical observation study of 2963 patients with wound samples from 910 DFUs, 916 VLUs, 767 DUs, and 370 non-healing surgical wounds, Wolcott et al. [42] demonstrated that *Staphylococcus* (Gram Positive) was the most frequently encountered genera. *S. aureus* and *S. epidermidis* were identified as the most abundant species in chronic wounds. Moreover, although bacterial diversity was independent of chronic wound type, *S. epidermidis* was found to be more prevalent in DFUs, and *Pseudomonas aeruginosa* (Gram Negative) exhibited a higher relative abundance overall in chronic wounds demonstrating biofilm formation. This study confirmed culture and electrophoresis techniques previously used by James et al. that identified Gram Positive cocci as the most abundant bacteria. However, in the case of biofilm formation, Gram Negative rods were the most abundant. Once again, both *Staphylococcus* and *Pseudomonas* were common in all chronic wound types [41]. Even though chronic wounds are naturally exposed to high levels of oxygenation, anaerobic bacteria have a stern presence in chronic wounds more so than acute wounds. Anaerobes such as *Fingelodia*, *Prevotella*, *Peptonipihlus*, *Peptostreptococcs*, and *Anaerococcus* have been identified as consistent microbial members of the chronic wound microbiome.

In terms of alpha-diversity, DFUs have been shown to be significantly less diverse then control skin both in species richness and evenness as measured by Chao and Shannon indices [47]. In addition, the beta diversity of microbial communities of control skin are significantly different than that of DFUs, as confirmed by permutational multivariate analysis of variance (PERMANOVA) [47]. Although Dowd et al. revealed *Corynebacterium* to be the most prevalent genera in 40 chronic DFUs, Gardiner et al. found *Staphylococcus* to be the most abundant genera in DFUs compared to controls [47,48]. Furthermore, the temporal stability of diversity measures is associated with healing outcomes. Although studies have shown that disease states are associated with less stability in the gut [49], destabilization may prove to be advantageous if the existing microbiota do not promote cutaneous wound healing. For example, Loesche et al. showed that DFUs were comprised of 4 community types, and the length of healing was associated with more transitions between these community types [50]. Moreover, they found that systemic antibiotics destabilized these pathogenic wound microbiomes resulting in faster wound healing, suggesting a role for antibiotics in targeting aberrant microbiome communities.

Although most studies focus on the presence of bacteria in chronic wounds, others have drawn attention to fungal communities. Fungal communities are common pathogens of various pedal infections and have now been identified as major players in the pathogenesis of non-healing wounds. Kalan et al. [51] identified a high prevalence of fungal communities in 100 non-healing DFUs. The most abundant fungal communities identified were Asomycota (*Cladosporidium herbarum* and *Candida albicans*) and Basidomycota (*Trichosporon* and *Rodhosporidum*). The researchers also demonstrated that administration of antibiotics, as well as the occurrence of clinical complications, were associated with increased fungal diversity, and classified pathogens were elevated in necrotic wounds. Clearly, more work needs to be done to fully grasp the role of fungi, and the interaction with other microbes in the context of wound healing [52]. Further understanding of these aspects of the microbiome may lead to better treatments for promoting wound healing.

### 3.2. Acute Wounds

While the bulk of clinical research on the role of various microbiomes on wound healing has focused on chronic wounds, acute wounds (e.g., burns, blunt traumas, and penetrating traumas) are also of interest. One clinical study demonstrated that cutaneous burns significantly altered microbial skin profiles, and adjacent skin of burn sites exhibited a more similar skin microbiome to that of the burn margin as opposed to that unburned controls [53]. Burn wounds in these patients revealed an increased abundance of thermophile microbes such as *Aeribacillus*, *Caldalkalibacilus*, and *Nesterenkonia* and decreased abundance of *Corynebacterium*, both in the wound center and the skin margin. Indeed, topical antibiotics have largely aided burn wound outcomes by effectively treating *Psuedomonas* colonization. However, changes in the cutaneous microbiome were also associated with post burn complications, with *Corynebacterium* demonstrating a positive correlation with burn wound infection, and *Staphylococcus* and *Propionibacterium* demonstrating a negative correlation with post burn infection [53]. Interestingly, Liu et al. also exhibited that skin dysbiosis is an expected consequence of burn injury that persists after healing [54]. In burn scars, community richness (an alpha diversity measure that represents the number of taxa in a population) was decreased even though these sites had an increase in bacterial diversity overall with an increased abundance of *Firmicutes* and *Staphylococcus* spp. compared to control skin [54]. However, all of these burn wounds demonstrated successful wound healing, and it was not examined if these changes in microbial composition adversely impacted wound healing rates.

Similar to burn injury, the wounds created as a result of blunt or penetrating traumas also exhibit changes in skin microbial composition and significant differences in beta diversity compared to controls [55,56]. The microbial composition of open fracture wounds is even associated with mechanism of injury [55]. The most dominant microbes in open fracture wounds and adjacent skin include *Staphylococcus*, *Corynebacterium*, *Streptococcus*, *Acinetobacter*, *Anaerococcus*, *Finegoldia*, and *Pseudomonas*. In this prospective observational study done by Bartow-McKenny et al., *Pseudomonas* was found to be the dominant species at the wound center on initial presentation to the emergency department (ED). Over time, the researchers found that the abundance of *Staphylococcus* greatly increased, and the abundance of *Pseudomonas* significantly decreased in the wound center. While the changes seen clinically provide information as to which bacteria may hold valuable diagnostic information, the section below examines molecular information from models that may be leveraged to modulate the microbiome and ensuing inflammation to aid in wound healing.

## 4. The Microbiome, Wound Healing and Inflammation: Mechanistic Insight from Model Systems

Mechanistic insight into the relationship of the altered microbiome and cutaneous inflammation has been aided by the use of preclinical studies, including animal models and in vitro systems. Although the ideal cutaneous wound model has not yet been established, all of these model systems may provide valuable mechanistic insight and act as screening tools for modulation of the human skin microbiome. In general, all of the models described have merit for examining different aspects of wound healing, and advantages of feasibility and throughput (e.g., in vitro models) may be initially used over more translatable in vivo models.

In vitro studies evaluating the impact of biofilms on chronic wound healing have included both epidermal and dermal investigations. Kirker et al. analyzed *S. aureus*-produced biofilms in a human keratinocyte wound model and found that the presence of biofilms resulted in significantly reduced scratch closure [57]. The Leiden epidermal model has also been used to study the impact of biofilms by creating an artificial stratified epithelium from human keratinocytes that demonstrates normal epidermal differentiation. Using this model, de Breji et al. demonstrated the ability for *Acinetobacter* spp. such as *A. baumanni* and *A. junii* to colonize and form biofilms on the most superficial layer of the epidermis (stratum corneum), which increased levels of IL-8 (a potent neutrophil chemoattractant) [58]. However, these organisms were not able to penetrate into the lower layers of the epidermis [58]. This same result was found with *S. aureus* as this microbe did not penetrate into the lower epidermal structures [59]. In addition, the dermis of the skin contains a wide number of cells essential for wound healing [60]. For example, biofilms have a detrimental effect on human dermal fibroblasts migration and ultimately result in cellular apoptosis [61]. Moreover, when compared to conditioned media of planktonic bacteria, *S. aureus* biofilm conditioned media increased the release of tumor necrosis factor α (TNF-α) and decreased the release of interleukin (IL)-6, matrix metalloproteinase (MMP-3) and VEGF from human dermal fibroblasts.

Rodents are often used for microbiome and wound healing research, however recent studies have brought into question the relationship between rodent and human inflammation [62,63]. In terms of wound healing, mice and rats have a thinner epidermis and dermis compared to humans, which primarily heals by contraction and not re-epithelization. However, many studies are able to overcome this difference with mechanical forces or other mechanisms [64]. Alternatively, large animals such as swine have also been used, as pigs have been regarded as the closest surrogate to human skin for similarities in structure and healing [65,66]. Recently, the porcine cutaneous microbiome has been shown to affect in vitro wound healing of human keratinocytes [67], and to be closer to humans on a relative scale [68]. However, dysbiosis and signaling pathways in, for example, atopic dermatitis have been shown to closely align with what is seen in humans [69]. Moreover, swine also have disadvantages when compared to rodents, including costs, handling, and the lack of genetic manipulability [65]. In short, the model chosen should be decided by the question being asked, as rodent models may adequately address, for example, clearance from infection at a lower cost than large animals.

To that end, murine wound models have reinforced our understanding that *S. aureus* and *S. epidermidis* biofilms delay wound re-epithelization in uninfected wounds, and this process has been shown to be influenced by quorum sensing. For example, Schierle et al. [70] demonstrated that when exposed to quorum sensing inhibitors (RNAIII inhibiting peptide), the cutaneous integrity of these murine wounds was restored, abolishing biofilm formation and obliterating bacterial bioburden [70]. Quorum sensing inhibitors represent a broad range of enzymes and compounds that are produced both naturally and synthetically [71]. For chronic wounds, agents that disrupt biofilm formation may be an attractive adjuvant to antibiotics to combat the development of antibiotic resistance.

The use of models to explore the effects of acute burn injury have also been studied. Local burn injury has been known to increase epithelial permeability in even unburned skin, exacerbating trans-epidermal water loss [72,73]. This raises the possibility of colonization/infection of adjacent “normal” skin, resulting in poorer healing outcomes. Plictha et al. demonstrated in both burned skin and distant unburned skin in C57 mice an increased abundance of anti-microbial peptides (AMPs) and proteases, and a decreased abundance of kallikrein peptidases (an expected response of skin inflammation) [72]. This effect elicits a decreased ability of the skin to inhibit the bacterial growth of common skin pathogens such as *S. aureus* and *P. aeruginosa.*

### Effects of Bacterial Colonization on Skin Inflammation and Cutaneous Homeostasis

Skin commensals influence a variety of cell signaling and homeostatic processes including keratinocyte proliferation, epithelial differentiation, and epidermal blood vessel growth. While elevations of microbial bioburden often result in infection, high diversity of skin commensals, as seen in healthy microbial colonization, are also involved in both the benign induction of the immune system and the attenuation of the immune response. For example, skin CD8+ T cells specifically elicited by *S. epidermidis* promote rapid keratinocyte progression via upregulation of toll-like receptors (TLR) and downstream modulation of TNF-α [74,75]. In addition, *S. epidermidis*’ production of lipoteichoic acid decreases cutaneous inflammation via TLR2 signaling [76]. The ability of *S. epidermidis* to modulate the innate immune response in non-infectious skin wounds coincides with its ability to accelerate wound healing in various skin models, and highlights the ability for bacterial products to reduce cutaneous inflammation.

Similarly, both *S. epidermidis* and the typically low-abundant *S. aureus* [77], induce expression of AMPs in human keratinocytes, ultimately benefiting skin by providing host protection from invasion of other pathogenic microorganisms [78,79]. An example of these AMPs is small cationic beta-defensin molecules (hBD) which are expressed in all human epithelial cells and play a role in epithelial differentiation [80]. Indeed, differential expression of these AMPs may confer the ability to amplify the innate immune response to commensal bacteria [79]. This appears to be in part, completed through known mechanistic pathways such as Nuclear Factor Kappa Beta (NF-κβ), Protein Kinase B (AKT), and TLRs. Although hBD-1 is continually expressed in human epithelial tissue, hBD-2 and hBD-3 are only expressed in skin tissue when stimulated by both pathogenic and commensal microbes including *S. epidermidis*, *S. aureus*, and *Group A streptococcus* spp. (GAS) [80]. The ability for commensals to facilitate production of these, exclusive yet beneficial, AMPs supports the idea that skin microbes play a vital role in epithelial differentiation and the maintenance of skin-barrier function; a role that cannot be fulfilled in their absence. The induced expression of these AMPs in the face of pathogens also renders these attractive targets for treatments to accelerate wound healing.

Although *S. aureus* is a normal commensal of human skin flora (albeit rare), overabundance of this microbe is associated with high rates of skin infection especially with production of superantigens (SAg) that have varying effects at the local and systemic levels. While exceptionally deleterious in high systemic concentrations, in small amounts, SAg production by *S. aureus* decreases local downstream production of interleukins like IL-17 and subsequent neutrophilic chemotactic factors in cutaneous tissue, resulting in decreased purulence of skin wounds and decreased skin inflammation compared to non-SAg producing strains [81,82]. The relatively low abundance of SAg production in these skin wounds might explain why the occurrence of the systemic cytokine storm seen in septic patients does not immediately develop in patients demonstrating *S. aureus* colonization. In addition, Secor et al. demonstrated that *S. aureus* biofilms resulted in significantly elevated keratinocyte cytokines, such as IL-1B, IL-6, chemokine ligand (CXCL)-8, CXCL-1, and TNF-α, demonstrating the potentially destructive effect these microbial products have on cutaneous inflammation [83].

The impact of *Pseudomonas* colonization on host epithelial tissue, once again highlights the diverse responses in integumentary cell signaling initiated by differing microbe levels. On initial contact with the skin, *Pseudomonas* localizes to the epithelial barrier of its host and at low levels this microbe not only accelerates epithelization, but also increases the rate of blood vessel growth in acute wounds through keratinocyte growth factor-1 and alteration of previously mentioned cytokines [84]. Similarly, as seen in a *Schmidtea mediterranea* wound model, a planaria organism known for its tissue regeneration abilities, *Pseudomonas* stimulates the TAK1/MKK/p38 signaling pathway, ultimately repressing cell apoptosis and improving wound healing outcomes in the absence of infection [85]. However, in the presence of infection, stimulation of TAK1/MKK/p38 by *Pseudomonas* spp. induced cell apoptosis and ultimately comprised tissue homeostasis, resulting in decreased frequency of neoblast cells and inhibition of tissue regeneration of amputated planaria fragments [85].

The varying effects of skin microbiota on cell signaling pathways suggest that small amounts of these potentially pathogenic microbes may in some cases aid rather than harm host cutaneous tissue regeneration. However, as mentioned earlier, in the absence of skin microbiota in rodents, wound healing was accelerated due to decreased abundance of bioactive markers secreted by recruited neutrophils [86]. This finding supports the idea that the process of inflammatory stimulation via bacterial colonization, although beneficial in many situations, may also adversely impact healing outcomes due to the increased abundance of proteases, reactive oxygen species, and other bioactive substances that delay wound healing. Another study supporting this theory found that nucleotide-binding oligomerization domain-containing protein 2 (NOD2), a pattern recognition receptor that recognizes bacterial peptidoglycans and consequently stimulates a host immune response, was found to be upregulated in non-healing murine cutaneous wounds [87]. At this point, it appears that various skin microbiota have both advantageous and detrimental effects on host cutaneous tissue in both uninjured and injured skin, which may dictate whether these microbes take on the role of skin commensal or skin pathogen. Taken together, preclinical models have identified a large number of potential molecular targets, summarized in Table 1.

## 5. Modulating the Microbiome: Clinical Implications for Wound Healing and Tissue Regeneration

**Figure 1:**
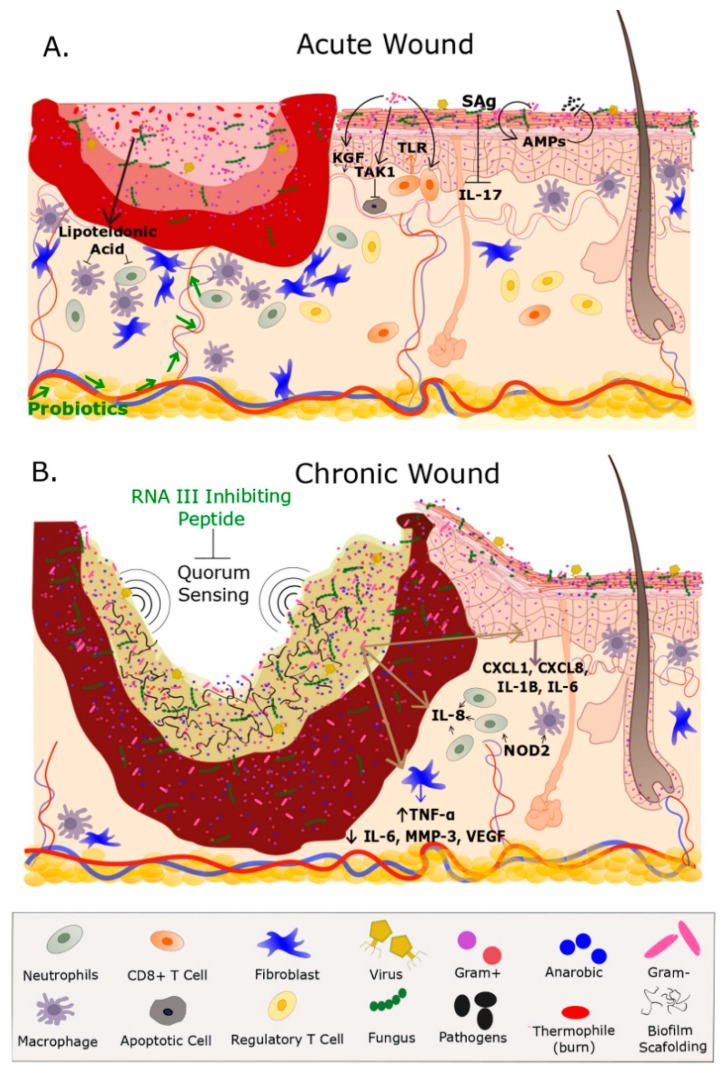
Microbiome, Wound Healing, and Wound Healing Impairment. (**A**) Acute wounds such as burns and open fractures contain a microbiome that may or not contain a vastly different population than what is on undamaged adjacent skin. The microbes present on an acute wound don’t necessarily impede the inflammatory response therefore allowing for the highly coordinated events that promote wound healing. Several fibroblast, and inflammatory cells such as macrophages, neutrophils, and T Cells are highly involved in the healing process. Production of lipoteichoic acid by *S. epidermidis* deceases inflammation. Keratinocytes express anti-microbial peptides (AMPs) in response to *S. epidermidis*, *S. aureus*, *Group A streptococcus* which provides protection for pathogenic bacterium. Supplementation with probiotics (e.g., *Lactobacillus reuteri*) accelerates wound healing although the mechanism is unknown. CD8+ T Cells in response to *S. epidermidis* enhance rapid keratinocyte progression via Toll-like receptor (TLR). Additionally, *Pseudomonas* accelerates epithelization and blood vessel growth through transforming growth factor beta-activated kinase 1 (TAK1) signaling. Overabundance of *S. aureus* produces superantigens (Sag) that are decreases interleukin (IL-17) and subsequently promoting wound healing. Note the greater numbers of healthy fibroblast, macrophages, and neutrophils near the wound bed and the intact vasculature that are essential in the healing process. Adjacent to the wound is undamaged (healthy) skin that contains macrophages, fibroblast, and a healthy ecological community of microorganisms that includes Gram Negatives, Gram Positives, fungi, and viruses within the epidermis and hair shaft; (**B**) chronic wounds contain a biofilm and a dense population of microorganisms which include anaerobic bacteria that obstruct wound healing by preventing topical antibiotics reaching the wound bed. Quorum sensing within the biofilm promotes biofilm formation, whereas exogenous topicals such as RNA inhibiting peptide inhibits biofilm formation. The biofilm elevates the expression of cytokines IL-1B, IL-6, chemokine ligand (CXCL) 1 and 8. Bacterium in the biofilm increase levels of IL-8 which is a potent neutrophil chemoattractant. Additionally, the biofilm increases levels of TNF-α, and decreases IL-6, MMP-3, and vascular endothelial growth factor (VEGF). Nucleotide-binding oligomerization domain-containing protein 2 (NOD2) stimulates a host response and is highly expressed in chronic wounds. The loss of vasculature to the wound bed further prevents the migration of immune-related factors and delivery of exogenous therapeutics. Fibroblast migration is impeded by biofilm formation Note the hyper-proliferative epidermis on the outer region of the wound bed in an attempt to epithelialize the wound. Although inflammation is present in chronic wounds the overall number of normal functioning fibroblasts and macrophages are low which further prevents healing. Green font represents an exogenous treatment for wound healing.

With rising concerns regarding antibiotic over-prescription and the development of pharmacologic resistance, finding alternative ways to modulate the microbiome in hopes of improving wound healing is of the utmost importance. Figure 1 illustrates some of the molecular targets identified, as well as other phenomenon that may be leveraged for accelerating wound healing. Clinical applications that target the microbiome to improve wound healing have already been demonstrated in the treatment of atopic dermatitis (AD). AD skin lesions often have a reduced abundance of skin commensals and, therefore, have resulted in a decreased production of AMPs [4,98,99]. One study found that reintroduction of AMPs in human subjects resulted in a decreased colonization of AD lesions by *S. aureus* [99]. Likewise, a multicenter study in AD patients in Germany found that treatment with a lotion containing *Lactobacillus johnsonii*, a common probiotic strain, resulted in significantly increased clinical improvement of AD lesions [98]. Gueniche et al. similarly found that topical application of *Vitreoscilla filiformis* improved the healing of skin lesions in AD patients [100].

Just as probiotics are widely commercially available for gastrointestinal issues, using probiotic organisms to restore the skin microbiome and improve cutaneous healing also has promise. Lactobacillus plantarum not only inhibits the production of elastase, biofilms, and acyl homoserine lactone by *Pseudomonas* spp. in vitro, but also demonstrated inhibition of *Pseudomonas* colonization and improved tissue repair in a burn mouse model [101]. In the presence of infection, *L. plantarum* containing probiotics reduced collagen accumulation in *Pseudomonas* infected burn wounds, ultimately decreasing the extent of scarring [102]. In chronic wounds, a randomized clinical trial revealed the potential advantageous healing properties of probiotics in DFUs with significant reductions in ulcer length and depth, in addition to improved glycemic control [103]. Similarly, Argenta et al. showed that *L. plantarum* showed an 80% decrease in mortality in a pseudomonas-infection porcine burn model [104]. Another possible treatment for hard to heal wounds that deserves further exploration is Honeybee lactic acid bacteria; when applied to chronic equine wounds this microbe promoted wound healing in all cases and inhibited the growth of all pathogens in vivo [105]. In line with this, honey alone has been shown to improve burn wound reepithelization rate despite a higher bacterial load when compared to 1% Silver Sulphadiazine in a porcine model [106]. Finally, in a recent meta-analysis, Kasatpibal et al. showed that symbiotic therapy reduces post-operative surgical site infection, pneumonia, sepsis, hospital stay and duration of antibiotic administration [107]. Taken together, initial evidence suggests that probiotics may be used in conjunction with antibiotics for the purposes of accelerated wound healing.

As mentioned earlier, short chain fatty acids (SCFAs) produced by skin commensals demonstrate vital anti-microbial properties and incorporation of these metabolites into the treatment of cutaneous wounds has high potential. When injected or applied topically, SCFAs such as acetic, butyric, and propionic acid suppress cutaneous inflammation by promoting skin T-regulatory cells (Tregs) in a histone acetylation dependent mechanism [108]. Furthermore, *S. epidermidis* increases the effect of probiotics through production of poly-ethylene glycol dimethacrylate, an inductor of SCFAs, effectively resulting in the decolonization of Methicillin Resistant *Staphylococcus Aureus* (MRSA) strain in infected skin wounds in rodents [109]. Poutahidis et al. revealed that supplementing the intestinal microbiome with the lactic acid bacteria *Lactobacillus reuteri* by adding it to drinking water, accelerated the cutaneous wound healing process in animals two-fold due to up-regulation of the neuropeptide hormone oxytocin [110]. This report showed that bacteria-induced oxytocin-activated FoxP3+CD25+host immune Tregs, decreased the inflammatory damage caused by the innate immune system and led to increased collagen deposition rates. Thus, both the cutaneous and gastrointestinal microbiome may influence wound healing.

Disrupting the formation of biofilms represents another potential therapeutic target to improve wound healing. As mentioned earlier, the application of quorum sensing inhibitors in a murine wound model resulted in the obliteration of *Staphylococcus* produced biofilms and decreased bacterial burden at the wound site [45]. Additional laboratory studies have supported the consensus that the inhibition of microbial quorum sensing is successful at limiting biofilm formation [111,112,113]. Another potential approach is the introduction of engineered synthetic peptides that possess unique anti-biofilm properties; these peptides are often derived from naturally produced host AMPs [114,115,116]. In a novel research study, Overhage et al. demonstrated that the naturally-produced human host defense peptide 11–37 not only inhibited the formation of *Pseudomonas* produced biofilms in-vitro, but also resulted in disruption of pre-grown bacterial biofilms [117]. This affect was achieved through influencing quorum sensing, decreasing bacterial adherence, and downregulating genes required for biofilm formation. Finally, a novel approach using predatory bacteria such as *Bdellovibrio bacteriovirus* has been shown to reduce biofilm formation [118]. These bacteria effectively destroy a large number of pathogens (especially Gram Negative species) and could have great implications for wound healing. However, clinical studies using these approaches have not yet been conducted, and further research in this field is needed in order to fully understand how the abrogation of biofilms may potentially improve wound healing.

## 6. Conclusions

Culture-independent methodological tools have only just begun to identify the vast array of microbial members that contribute to the cutaneous microbiome. In this review, we chose to focus primarily on better known bacterial species since they currently have a greater influence on clinical practice. However, with the advent of next-generation sequencing technology we are apt to discover even more species that co-exist in and on our bodies. Yet, due to the limitations of cell culture, the implications of these novel species are unclear. One of the major hurdles facing this field is determining the effect of the microbiome on our health both at the level of the individual species and as vast polymicrobial communities. Skin commensals such as *Staphylococcus*, *Streptococcus*, *Pseudomonas*, *Corynebacterium*, and various anaerobes have both advantageous and detrimental effects, depending on properties such as load and the hosts’ cutaneous environment. Skin commensals provide benign tonic stimulation to the host’s immune system and deter invasion of other pathogenic microbes; however, in the presence of tissue injury, loss of microbial diversity often results in prolonged inflammation, which can delay wound healing. This intimate relationship with host inflammation may reveal therapeutic targets that could circumvent or augment antibiotics, such as the introduction of anti-microbial peptides through topical solutions. Furthermore, variables such as wound etiology, location, and health status of the patient help dictate the dynamic constituents of skin commensals. Recent research has allowed us to target the microbiome in various ways to improve wound healing; restoration of the skin microbiome with Lactobacillus containing probiotics or applying agents that disrupt biofilms may improve wound healing outcomes. Although substantial progress has been made in microbiome research, further studies are needed in order to elucidate how the microbiome impacts wound healing, and vice versa. In addition, while the mycobiome and virome influence wound healing, they represent a potential untapped resource in the understanding and promoting of wound healing. Ultimately, the goal is to leverage the knowledge gained on the skin microbiome to promote the healing of both acute and chronic wounds.

## Figures and Tables

**Table 1:** Skin commensals have varying effects on host cutaneous tissue and are associated with a variety of cell signaling pathways.

Bacteria	Positive Effects	Negative Effects	Associated Signaling Pathways
*Staphylococcus epidermidis*	Stimulates keratinocyte production of host AMPs (hBD3, RNase7) [22,74,75,88] Induces CD8+ T and IL-17A+ T cells [79] Enhances innate barrier immunity and limits pathogen invasion in absence of inflammation [6,74,80,89]	Occasionally pathogenic Implicated in production of biofilms [79,90,91,92,93]	NF-κB [74,76,89] TRAF1 [7] TLR2/CD36/CD14-p38, MAPK [7] EGFR TRAP [70]
*Staphylococcus aureus*	At a local level, super antigen production results in less skin inflammation and purulence due to decreased production of exotoxins and neutrophilic chemotactic factors [81] Amplifies innate immune response of skin via production of AMPs (hBD-3, hBD2, LL-37, RNAse7) [78,79]	Usually pathogenic Implicated in production of biofilms and delayed wound healing in chronic wounds [91,92] Super antigen production elicits robust activation of immune system [81]	TRAP [70,94] phosphatidylinositol 3-kinase/AKT NF/kB ERK TLR-2 [9]
*Group A streptococcus (GAS)*	Stimulates production of AMPs, promote epithelial differentiation [95] Activates plasminogen which promotes Keratinocyte chemotaxis and potential re-epithelization of wounds [95]	Usually pathogenic Express proteases which prevent neutrophil recruitment [79,96,97] Produces hyaluronidase which allows bacteria migration through host Extracellular matrix [7] Common cause of superficial and deep skin infections i.e., impetigo, erysipelas, cellulitis [6]	NF-κB/p65 [80]
*Pseudomonas aeruginosa*	Accelerates epithelialization and neovascularization in acute wounds Suppresses staphylococcal pathogens in polymicrobial wounds [84]	Usually pathogenic Implicated in production of biofilms and delayed wound healing in chronic wounds [90,91,92,93]	Nod2 [87] TAK1/MKK/p38 [85]
*Corynebacterium jeikeium*	Manganese acquisition and production of superoxide dismutase result in host epidermal protection from free radical oxygen species (ROS) [5]	Occasionally pathogenic Common cause of nosocomial skin infections [95]	N/A
*Propionibacteria*	Production of bacteriocins protect sebaceous ducts from other pathogenic inhabitants [77] induces expression of TLR2 and TLR4 in keratinocytes71	Occasionally pathogenic Overabundance associated with development of Acne [5]	N/A

## Abbreviations

AKT/PKB	Protein Kinase B
AMP	Anti-Microbial Peptide
CXCL	Chemokine Ligand
DFU	Diabetic Foot Ulcer
DNA	Deoxyribonucleic Acid
DU	Decubitus Ulcer
EGF ^®^	Epidermal Growth Factor (Receptor)
ERK	Extracellular Signal Regulated Kinase
FOXP3	Forkhead Box P3 Protein
GAS	Group A *Streptococcus*
hBD	Human Beta Defense Protein
HPV	Human Papilloma Virus
IL	Interleukin
KGF	Keratinocyte Growth Factor
MAP	Mitogen Activated Pathway
MAPK	Mitogen Activated Pathway Kinase
MKK	Mitogen Activated Pathway Kinase Kinase
MMP	Matrix Metalloproteinase
MRSA	Methicillin Resistant *Staphylococcus aureus*
NF-κβ	Nuclear Factor Kappa Beta
NOD	Nucleotide-binding oligomerization domain-containing protein
PI3K	phosphatidylinositol 3-kinase
rDNA	Ribosomal Deoxyribonucleic Acid
RegIIIy	Regenerating Islet Derived Protein Gamma
RNA	Ribonucleic Acid
Rnase	Ribonuclease
ROS	Reactive Oxygen Species
Sag	Superantigen
SCFA	Short Chain Fatty Acid
TAK	Transforming Growth Factor (TGF) F β-activated kinase
TGF	Transforming Growth Factor
TLR	Toll Like Receptor
TNF	Tumor Necrosis Factor
TRAF	Tumor Necrosis Factor (TNF) receptor-associated factor
TRAP	Target of RNAIII activating protein
Treg	T regulatory Cell
VEGF	Vascular Endothelial Growth Factor
VLU	Venous Leg Ulcer
